# Domesticated, Genetically Engineered, and Wild Plant Relatives Exhibit Unintended Phenotypic Differences: A Comparative Meta-Analysis Profiling Rice, Canola, Maize, Sunflower, and Pumpkin

**DOI:** 10.3389/fpls.2017.02030

**Published:** 2017-12-05

**Authors:** Alejandra Hernández-Terán, Ana Wegier, Mariana Benítez, Rafael Lira, Ana E. Escalante

**Affiliations:** ^1^Laboratorio Nacional de Ciencias de la Sostenibilidad, Instituto de Ecología, Universidad Nacional Autónoma de México, Mexico City, Mexico; ^2^Laboratorio de Genética de la Conservación, Jardín Botánico, Instituto de Biología, Universidad Nacional Autónoma de México, Mexico City, Mexico; ^3^Centro de Ciencias de la Complejidad (C3), Universidad Nacional Autónoma de México, Mexico City, Mexico; ^4^Facultad de Estudios Superiores Iztacala, Universidad Nacional Autónoma de México, Mexico City, Mexico

**Keywords:** genotype–phenotype, unintended phenotypic effects, phenotypic profiling, *Oryza sativa*, *Brassica napus*, *Helianthus annuus*, *Zea mays*, *Cucurbita pepo*

## Abstract

Agronomic management of plants is a powerful evolutionary force acting on their populations. The management of cultivated plants is carried out by the traditional process of human selection or plant breeding and, more recently, by the technologies used in genetic engineering (GE). Even though crop modification through GE is aimed at specific traits, it is possible that other non-target traits can be affected by genetic modification due to the complex regulatory processes of plant metabolism and development. In this study, we conducted a meta-analysis profiling the phenotypic consequences of plant breeding and GE, and compared modified cultivars with wild relatives in five crops of global economic and cultural importance: rice, maize, canola, sunflower, and pumpkin. For these five species, we analyzed the literature with documentation of phenotypic traits that are potentially related to fitness for the same species in comparable conditions. The information was analyzed to evaluate whether the different processes of modification had influenced the phenotype in such a way as to cause statistical differences in the state of specific phenotypic traits or grouping of the organisms depending on their genetic origin [wild, domesticated with genetic engineering (domGE), and domesticated without genetic engineering (domNGE)]. In addition, we tested the hypothesis that, given that transgenic plants are a construct designed to impact, in many cases, a single trait of the plant (e.g., lepidopteran resistance), the phenotypic differences between domGE and domNGE would be either less (or inexistent) than between the wild and domesticated relatives (either domGE or domNGE). We conclude that (1) genetic modification (either by selective breeding or GE) can be traced phenotypically when comparing wild relatives with their domesticated relatives (domGE and domNGE) and (2) the existence and the magnitude of the phenotypic differences between domGE and domNGE of the same crop suggest consequences of genetic modification beyond the target trait(s).

## Introduction

Plant domestication and the phenotypic modifications it produces have a long history with humans and have involved practices ranging from traditional management to genetic engineering (GE). The effectiveness of traditional practices, or human selection, is possible because the selected traits have a genetic basis that are phenotypically expressed in particular agroecological and cultural environments (Gepts, 2004; Meyer and Purugganan, 2013). Consequently, domestication processes, either with or without GE, may have important evolutionary effects in cultivated plants (Abbo et al., 2014; Hake and Ross-Ibarra, 2015). Genetically modified crops are also domesticated plants, since the genetic modifications are performed in isogenic lines of the crop of interest (Setlow, 1991). Nonetheless, these domestication processes are qualitatively different. On the one hand, in traditional plant breeding new genetic combinations are, in general, obtained by sexual crosses between individuals of the same species. In GE, on the other hand, DNA sequences (of potentially non-related organisms) are inserted into the crop of interest via bioballistics, *Bacillus thuringiensis* (*Bt* crops) (Agrawal et al., 1999; Nodari and Guerra, 2001) and other novel techniques (e.g., CRISPR, RNA_i_) (McManus and Sharp, 2002; Gaj et al., 2013). Thus, the main differences between the two genetic modification techniques involved in domesticated plants are (i) the origin of the novel or foreign DNA that is incorporated in the modified organism, and (ii) the procedure to accomplish such incorporation (Gepts, 2001; Nodari and Guerra, 2001).

Agronomic modification via human selection, domestication without genetic engineering (domNGE), or through genetic engineering (domGE) have phenotypic effects that may not correspond, in magnitude, to the associated genetic changes (Burke et al., 2007). Some of these phenotypic changes are unintended and are usually unrelated to the target traits (Filipecki and Malepszy, 2006). Some studies have attributed these unintended phenotypes to pleiotropic effects in which certain phenotypic traits may be linked and affected by the genetic modification of another trait (Filipecki and Malepszy, 2006), as well as to bottlenecks, selective sweeps, phenotypic plasticity, or gene × environment (G × E) interactions (Remington et al., 2001; Pozzi et al., 2004; Gunasekera et al., 2006; Doust et al., 2014). This phenomena, in which the domesticated organisms show phenotypes that do not correspond to the target traits of domestication, has been documented in many crops, such as potato (*Solanum tuberosum*), soybean (*Glycine max*), and wheat (*Triticum aestivum)* (Dale and McPartlan, 1992; Gepts, 2004; Lenser, 2013). Some of these modified non-target traits have been found to be related to species fitness, which in turn can have a direct impact in the evolution of the plants in potentially unexpected ways (Meyer and Purugganan, 2013).

The unintended phenotypic effects and their evolutionary (and potentially ecological) consequences are of particular relevance if we consider that most of the modifications are done in economically important crops. As such, unintended changes in phenotypes have been observed in crops that are key for global food production, such as rice (*Oryza sativa*), canola (*Brassica napus*), sunflower (*Helianthus annuus*), pumpkin (*Cucurbita pepo*), and maize (*Zea mays*) (Snow et al., 1998; Spencer and Snow, 2001; Halfhill et al., 2005; Guadagnuolo et al., 2007; Cao et al., 2009). Moreover, for cases such as maize, pumpkin, and rice, their cultivation represents important sources of cultural value that involve practices related to their diversification, achieved through the traditional selection of ancestral populations (Purugganan and Fuller, 2009; Chen et al., 2015), and represent an important cultural and genetic repository (Altieri and Merrick, 1987).

Moreover, in the context of food security under climate change and high uncertainty scenarios, *in situ* conservation of agrobiodiversity is of key importance, including not only phenotypic and genetic diversity, but also the accompanying management practices and the environmental context that allows future adaptation (including wild relatives) (Kahane et al., 2013). Therefore, and beyond the merely evolutionary consequences of unintended phenotypic changes, agrobiodiversity studies that look into specific trait changes can help improve protocols of biosafety and risk assessment (Smyth and Mchughen, 2008).

Although the phenomena of the unintended effects of genetic modification have been widely reported, these observations are the product of many individual studies. Thus, we propose a meta-analysis profiling approach in order to perform an unbiased analysis with high statistical power. Meta-analysis profiling allows for the integration of large quantities of data in order to identify patterns among different studies that share a common theoretical framework, but that have been conducted independently (Fiehn et al., 2000). This approach represents a valuable tool that has been used to identify patterns in plant functional genomics (Fiehn et al., 2000) and in phenotypic traits related to growth in plants (Kjemtrup et al., 2003). In the present study, we aimed to profile as many observations as possible into a meta-analysis of the phenotypic consequences of agronomic improvement in five economically and culturally important crops: rice, canola, sunflower, pumpkin, and maize. For the analysis, we included functional phenotypic traits that are potentially related to plant fitness, independently of whether these traits were modified through traditional practices (domNGE) or genetic engineering (domGE), so we could determine whether there were unintended phenotypic and thus evolutionary consequences. This profile includes 120 scientific publications (110 papers and 10 theses), which cover the period 1990–2017.

## Materials and Methods

### Data Collection

In order to determine whether genetic modifications have unintended phenotypic consequences in plants, we identified suitable studies for our analysis by looking for articles published in agricultural and ecological journals, as well as in the thesis database for the National Autonomous University of Mexico (UNAM) for the case of maize. We focused on five of world’s most economically important species: rice, canola, sunflower, pumpkin, and maize. We searched for information in the Scopus^®^, GoogleScholar^®^, and UNAM theses databases. For Scopus^®^ and GoogleScholar^®^ databases, we employed Boolean operators for each crop, such as “Cucurbita [AND] wild [OR] domesticated [OR] GMO [AND] fitness.”

To be included in the database, all publications had to satisfy three selection criteria: (1) An estimate of plant fitness between wild relatives and domesticated varieties with and without GE must have been measured; (2) Tests must have been performed under conditions in which the agent of selection was absent; and (3) The genetic background must have been controlled to minimize differences affecting the fitness traits being measured. In cases where experiments included extreme biotic and/or abiotic conditions (e.g., soil fertilization, heat, drought), only the data of the controls were used, since we considered these treatments as perturbations and not as natural environmental variation. In the case of maize, we also used information from thesis reports in which a yield comparison between wild relatives and domesticated relatives was performed. All thesis reports had gone through a peer review process [Reglamento General de Estudios de Posgrado (RGEP), UNAM]^1^ and were obtained from the National Autonomous University of Mexico theses database. We applied these criteria rigorously, rejecting hundreds of comparisons that did not satisfy all of them.

Of all the available information, only 110 articles and 10 theses, representing 990 comparisons of wild relatives and domesticated varieties with and without GE of the five species were incorporated into our dataset. The comparison for each genotype and the number of analyzed publications by crop are shown in **Table 1**. The data reported in the articles were collected for the period 1990–2017, representing the timeframe of the first release of genetically modified organisms to date. Although the available literature sometimes reports more phenotypic traits, only six were chosen in the analysis presented here: height (cm), number of flowers, days to flowering, number of seeds, pollen viability (%), and number of fruits. Those traits were chosen because they are functional traits that have potential impacts in survival and reproduction of the plants (Dafni and Firmage, 2000; Saatkamp et al., 2011; Huang et al., 2016; Williams and Mazer, 2016), besides their availability in most of the published studies. The full dataset can be found in the **Supplementary Data Sheet S1**.

**Table 1:** Comparisons for each category [wild, domesticated without genetic engineering (domNGE), domesticated with genetic engineering (domGE)] and total number of publications analyzed in each crop (*N* = number of reviewed publications).

Crop	Wild	domNGE	domGE	Comparisons	*N*
Rice	64	57	98	219	33
Canola	34	114	52	200	22
Sunflower	27	81	11	119	11
Pumpkin	19	37	33	89	19
Maize	54	254	58	366	35

### Data Analysis

To standardize data from different traits, we followed a procedure based on Song et al. (2004). The method consists in taking all the values of a single trait from low to high, and normalizing between zero and one. Outlier data points were identified using the Viechtbauer and Cheung (2010) approach. In this approach, a multivariate detection method (Cook’s distance) is used to calculate the distance among all data points, and then the data points that do not fall into the general model are identified as “influential data points” or outliers. Given the potential biological meaning of outliers (extreme phenotypes), we decided to investigate the experimental origin of each data point before removing it from the database. We considered that the only biologically meaningful outliers would be those which corresponded to common garden experiments of the domGE with their domNGE isogenic lines, in which case, and despite the outlier category of the data point with respect to the general model, we did not remove these data points from the rest of the analysis. This process was performed for all traits and all crops. As we mentioned before, in most cases the genetic modification is performed in domesticated lines, therefore we decided to separate the three categories in all crops with the labels: “wild” for wild relatives, “domNGE” for domesticated organisms that have not gone through a GE process, and “domGE” for those which have been genetically modified to show new traits.

To determine statistical differences among wild, domNGE, and domGE categories within species, we used a Generalized Linear Model (GLM). In the cases where the *p*-value was less than 0.05, we carried out a Glht (Tukey) as a *post hoc* test in the *R* Multcomp package (Hothorn et al., 2008). A graphic representation of the data was constructed as a Spider Chart using *R* Fsmb package (Nakazawa, 2014). In addition, to determine differences between categories (wild, domNGE, and domGE) within species, we conducted a Discriminant Analysis (DA) with the *R* MASS package (Venables and Ripley, 2002) using the genotypes as categories and the values of each trait as predictor variables. To test the significance of differences between categories of the DA per crop, we conducted a follow-up Multivariate Analysis of Variance (MANOVA). Finally, we delimited groupings by drawing 95% confidence interval ellipses around the centroids using the ggplot2 R package (Wickham, 2009). All the analyses were conducted in *R* program (version 1.17.15) (R Core Team, 2013) and all the scripts utilized for the analyses are available online at https://github.com/LANCIS-escalante-lab/plant_phenotype_metaanalysis.

## Results

The results of the 990 comparisons show significant phenotypic differences among the three categories (wild, domNGE, and domGE) for almost all of the analyzed crops and the majority of the traits. With regard to outlier management, the number of points that lie outside the normal distribution was significantly less than the total number of comparisons for each crop. In the case of canola, the outliers represent 2% of the total comparisons, for sunflower 1.8%, for rice 4.3%, and for maize 12%. In the case of pumpkin, we did not find any outliers.

**FIGURE 1:**
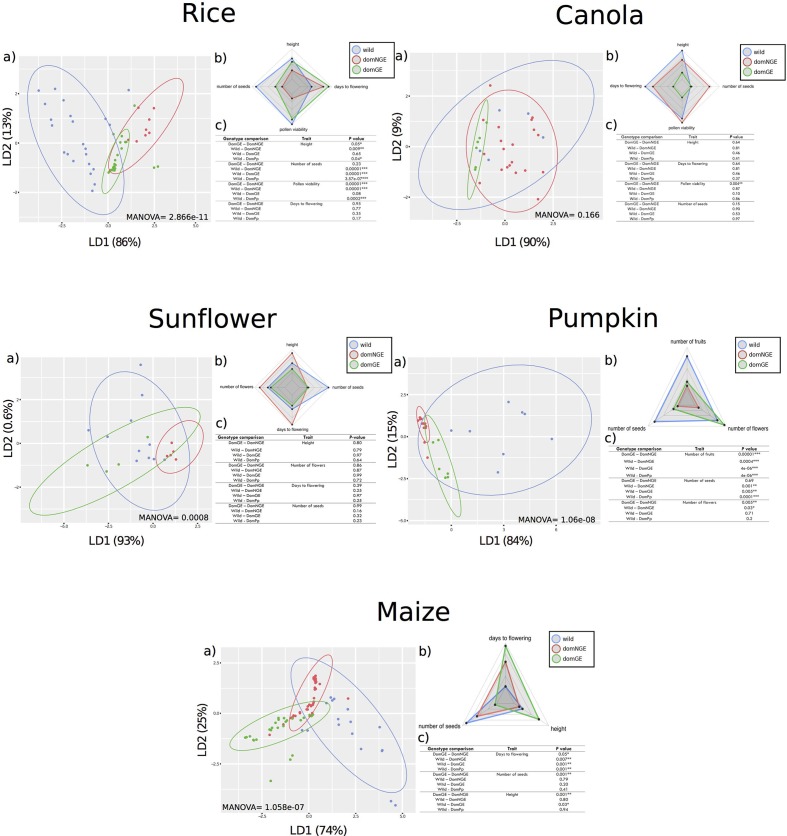
Phenotypic differences between wild and domesticated with and without genetic engineering (GE) in five crops. In all panels: (a) Discriminant analysis (DA), (b) Spider-Chart of the means of each analyzed trait, and (c) results of pairwise comparisons with Generalized Linear Model (GLM). In all (c) “DomPp” = both domesticated populations (GE and NGE). In all panels: “blue” wild relatives, “red” domesticated without GE, and “green” domesticated with GE. ^∗^*p* < 0.01; ^∗∗^*p* < 0.001; ^∗∗∗^*p* < 0.0001.

The differences between wild relatives and the domesticated categories (either domNGE or domGE) were expected, but unexpected differentiation between the domesticated categories (domNGE and domGE) was also observed in the analyzed traits (which were not the target of selection or genetic modification). Since the proportion of outliers within the dataset is relatively small, this general pattern observed in the results is maintained regardless the outlier treatment, with only some specific differences per crop (**Figure 1** and Supplementary Figure S1).

### Phenotypic Variation Can Be Identified As Wild, domNGE, and domGE

Through the DA of the phenotypic traits of all crops (height, days to flowering, number of seeds, pollen viability, number of flowers, and number of fruits), we found a clear distinction of phenotypic variation in three groups, which correspond with the wild, domNGE, and domGE categories [**Figure 1**, all (a) panels]. These three groups are different in size, position, and/or direction along the axes of the DA. In some cases, the overlapping of the groups is larger than in others. For instance, in canola, although the groups can be differentiated, the overlapping of the three groups is the largest compared with the other analyzed crops (MANOVA *F*_(2,52)_ = 1.541, *p* = 0.166), while in maize (MANOVA *F*_(2,116)_ = 8.5571, *p* = 1.058e^-07^) and rice (MANOVA *F*_(2,100)_ = 11.284, *p* = 2.868e^-11^) the overlapping is the smallest of all, with pumpkin (MANOVA *F*_(2,46)_ = 13.357, *p* = 1.066e^-08^) and sunflower (MANOVA *F*_(2,48)_ = 4.1348, *p* = 0.00081) in an intermediate range of overlapping [**Figure 1**, all (a) panels]. Moreover, the percentage of variation explained by the discriminant axes varies considerably among crops, with the most extreme cases being maize and sunflower. For maize, the total phenotypic variation is distributed in the two discriminant axes (LD1 = 74%; LD2 = 25%), while in sunflower and canola, the variation is mainly explained by LD1 (93 and 90%, respectively). A more detailed analysis of the DA results shows that the dispersion of the phenotypic variants within groups is, in most cases, larger in wild groups than in domesticated ones (domNGE and domGE) [**Figure 1**, all (a) panels]. The only case where the phenotypic variation found in the wild group was less than that found in the domGE groups was in sunflower.

### Variation in Phenotypic Traits Changes from Wild to Domesticated Populations

The DA results show a change in the direction of variation between wild and domesticated (domNGE and domGE) categories [**Figure 1**, all (a) panels]. This observation implies that the traits that define the phenotypic variation within each group are different, at least between wild and domesticated categories [**Figure 1**, all (b) panels]. In fact, in almost all the cases, the phenotypic variation of the domesticated groups goes in the same direction, while the wild group is almost orthogonal, and more evenly distributed between the two axes. This observation holds for all of the five analyzed crops.

The weight of the different traits in the resulting grouping per crop is provided by the associated coefficients of each discriminant function (Supplementary Table S1). Thus, it is possible to identify the traits that are statistically more important in the observed differences among groups. For rice, “height” is the trait with the highest coefficient for LD1 and “days to flowering” for LD2. For canola, “number of seeds” is the trait with the highest coefficient for LD1 and “height” for LD2. For sunflower, “days to flowering” has the highest value for LD1 and “number of seeds” for LD2. For pumpkin, “number of fruits” and “number of seeds” were the traits with highest values for LD1 and LD2, respectively. Finally, for maize, “height” is the trait with the highest value for both LDs.

The GLM analysis identifies the traits that explain pairwise differences in phenotypic variation among groups and the results are shown in the (c) panels of **Figure 1**. For instance, for sunflower none of the four analyzed traits show significant differences between wild and domesticated populations. In contrast, for maize, pumpkin, and rice almost all of the analyzed traits show significant differences (days to flowering, number of seeds and height for maize, number of fruits, number of seeds and number of flowers for pumpkin, and height, number of seeds, and pollen viability for rice) [**Figure 1**, (c) panels].

### Changes in Phenotypic Variation among Wild, domNGE, and domGE

The normal sequence of reduction of genetic (and potentially phenotypic) variation in the process of domestication and human interventions suggests that wild relative populations represent the largest pool of diversity, which is then reduced during domestication and genetic modification through GE (Flint-garcia, 2013). Moreover, since GE constructs start from isogenic lines (representing the domNGE), and since the modifications are allegedly directed to modify specific phenotypic traits (not included in the present analysis), it was expected that the phenotypic variation of the analyzed populations would be a sequence of subgrouping and reduced phenotypic variation going from wild to domNGE and finally domGE. However, through the DA and GLM analyses [**Figure 1**, panels (a) and (c), respectively], we find evidence that, overall, supports these expectations for the comparison of wild and domesticated categories (domNGE and domGE), but that do not hold for the comparisons between domesticated categories (domNGE vs. domGE). A graphical representation of these results, showing only mean values for all traits and populations, is found on **Figure 1**, (b) panels.

Regarding the comparison between the wild and domesticated (domNGE and domGE) categories, we observe that only canola fits the expectation of subgrouping. In contrast, regarding the reduced phenotypic variation of domesticated groups compared with their wild relatives, 4/5 analyzed crops fit the expectation (sunflower was the exception). These four crops (rice, canola, pumpkin, and maize) show that, although the phenotypic variation is reduced in the domesticated groups, this is not a subgroup within the wild group. The exceptional case, of sunflower, shows that domGE groups have increased phenotypic variation compared with both domNGE and wild relative groups. The results of the GLM [**Figure 1**, (c) panels], which investigates pairwise differences between wild and domesticated groups (Wild-DomPp), show that rice, pumpkin, and maize have statistically significant differences for almost all traits.

Regarding the comparison of domNGE vs. domGE, we observe that, on the one hand, rice and canola are cases in which the results show some subgrouping of domGE within domNGE populations. On the other hand, maize, sunflower, and pumpkin represent almost the opposite scenario, with almost no overlap, nor subgrouping of the domGE within domNGE populations. Regarding the expectation of reduced phenotypic variation in domNGE, we observe a case of increased phenotypic variation, and specifically we found that domGE groups of sunflower have more variation than their domNGE relatives. Moreover, we also found statistically significant differences in the pairwise comparisons between domNGE and domGE groups in almost all crops and traits [**Figure 1**, (c) panels]. For rice, we found differences between domesticated groups in “pollen viability” and “height;” for canola we found differences in “pollen viability;” for pumpkin the differences were found in “number of fruits” and “flowers;” finally, in maize we found statistical differences in “days to flowering,” “height,” and “number of seeds.” Overall, these results suggest unintended phenotypic effects, and no consistency in the specific traits that change due to human interventions in wild populations, either through domestication or GE modifications.

## Discussion

Human interventions in plants of economic, cultural, or nutritional importance via traditional practices (domestication) and, more recently, via GE have a long history in crop management. Despite the major importance of the consequences of these human-driven interventions in crops, no systematic investigation of the actual consequences in plant populations exists. In this study, we conducted a meta-analysis profiling the phenotypic consequences on non-target traits that domestication and GE have had for five global important crops, and here we discuss the potential causes and implications of our observations.

The nature of any meta-analysis implies a large amount of data points or measurements that may correspond to many different individual studies, with different environmental conditions and subject to different sources of error. Given this, it is important to consider carefully both the meaning and treatment of outlier data points, and the implications in the results of the implicit environmental variation. On the one hand, in this study we only removed those outliers that did not correspond to common garden experiments, and thus had biological relevance; in this case the occurrence of extreme phenotypes or big evolutionary leaps [possible “hopeful monsters” (Goldschmidt, 1940; Gould, 1977)]. Nonetheless, of all the comparisons in our analysis, only 3.2% were identified as outliers and, among these, 1% was “true” outliers (not coming from common garden experiments). Moreover, a very limited number of traits of the phenotype were included in the analyses, which precludes us from making major biological or evolutionary inferences about the identified outliers in the different crops, although we recognize the relevance of a more in-depth investigation of those outliers in the evolution of domesticated plants. On the other hand, and regarding the contribution of environmental variation to our overall results, given that different data points correspond to experiments conducted in different environmental conditions, it is not possible to rule out that the observed variation in phenotypes corresponds (in some proportion) to the variation in environmental conditions, and therefore caution should be taken in attributing the observations solely to the genotypic background of populations.

### Direction and Magnitude of Phenotypic Variation Changes between Wild and Human-Modified Plant Populations

The differentiation of wild and domesticated populations was expected due to the genetic changes that occur in the evolutionary process of domestication. The genetic changes can be the result of genome level modifications (e.g., genetic bottlenecks), but also can be the result of more localized effects associated with genetic linkage of selected regions (e.g., selective sweeps) (Gepts, 2004, 2014; Pozzi et al., 2004). The phenotypic and genetic variation of wild populations represents the pool from which some variants are selected, thus reducing the original variation via domestication and GE of isogenic domesticated lines (Innan and Kim, 2004). This phenomenon of paired phenotypic variation reduction due to genetic bottlenecks has in fact been described in previous studies with the same crops in this study and others (Miller and Tanksley, 1990; Tenaillon et al., 2004; Stupar, 2010). Nonetheless, we found a notable exception in sunflower, in which variation increases from that observed in the wild relatives. This exceptional case could be explained by the large phenotypic and genetic variation found in the continuum of landraces, hybrids, and genetically modified organisms that increases the phenotypic amplitude in the domesticated populations (McAssey et al., 2016).

Moreover, for most cases, we also observe change in the axis of the variation that could be attributed to the selection of certain variants for the target traits that will then vary in another direction, carrying along linked phenotypic variation in non-target traits. Altogether, the expected reduction of phenotypic variation and the change in the direction of this variation is in accordance with the concept of the domestication syndrome (Doebley et al., 2006; Meyer et al., 2012). However, we did not find consistency in the specific traits that varied among the three categories (wild, domNGE, and domGE). Potential explanations for this lack of shared traits in the differentiation of populations among crops could be, on the one hand, that although some phenotypic and general traits have been linked to the domestication syndrome, there are many others that are particular to each crop which are associated with specific aspects of their biology. For example, one of the most extreme cases of domestication is maize, where the phenotypic similarities between teosinte (wild ancestor) and contemporary maize are very small. The most important traits that define the domestication syndrome in maize are the change in the number and arrangement of ears and the presence of shorter lateral branches (Wills and Burke, 2007). Nevertheless, in many crops difficulties and ambiguities still exist in defining the domestication syndrome. One good example of such difficulties is Asian rice, in which the high levels of introgression between wild relatives and domesticated populations have caused genetic exchange that makes it difficult to identify the phenotypic traits that distinguish wild from domesticated populations (Vaughan et al., 2008). On the other hand, during the domestication process via selection, the phenotypic targets (or traits) are different for different crops. For instance, while in the case of rice the target of selection is the number of grains (seeds) (Vaughan et al., 2008), in the case of pumpkin, it is size and number of fruits (Meyer et al., 2012). In the same sense, GE of crops has different goals, thus different traits are introduced to different crops. For example, for canola a broad range of traits added through GE exists, such as insect resistance (Lepidopteran), herbicide tolerance (glyphosate/glufosinate), and virus resistance, while in pumpkin, the most frequent genetic transformation is focused on mosaic virus resistance (*ZYMV*) (Supplementary Table S2).

### Unexpected Phenotypic Changes in Human-Modified Plant Populations – Changes in Non-target Traits

As mentioned before, given that the GE constructs start from isogenic lines (represented here as domNGE), and that the modifications are allegedly directed to modify specific phenotypic traits not included in the present analysis, it was expected that the phenotypic variation of the domGE would be a subgroup of that in the domNGE group. This expectation is based on the premise that GE works usually with foreign DNA in order to introduce traits that are not present in the species, and this is performed in isogenic hybrid lines; thus, theoretically, the only differences between a Genetically Modified Organism (GMO) and its isogenic line will be the added trait(s) (Cellini et al., 2004). However, we did not find evidence that supports this expectation, suggesting unintended phenotypic effects of GE modifications. Specifically, we identified the most dramatic cases in rice, pumpkin, and maize, where almost all analyzed traits differ statistically between domNGE and domGE categories.

Generally, the intended effects of a genetic modification refer to a specific phenotype. But the transgene may also impart a range of phenotypes that constitute the unintended effects of the transgene. These new (unintended) phenotypes can appear due to the interaction of the transgenes with another genes (pleiotropic effects) (Rijpkema et al., 2007) or by position effects; thus, these unintended phenotypes are usually unpredictable (Miki et al., 2009). The cases in which the transgene, due to genetic interactions, causes unexpected phenotypes have been seen in canola (Légère, 2005), sunflower (Snow et al., 2003), rice (Chen et al., 2006), and maize (Guadagnuolo et al., 2007) among others. Although we intended to control the data for major environmental variation in the comparisons, we cannot rule out phenotypic plasticity due to GxE interactions that may be introducing a confounding effect in the observations. Moreover, these phenotypic differences between closely related (genetically) organisms can also be associated with other factors that depend on the origin and specific context of domestication that may end up in different phenotypic scenarios, causing phenotypic diversity between organisms of the same species (Gepts, 2001).

In addition to pleiotropy, other phenomena related to the genetic modification, such as position effects, that result from non-directed insertions of DNA fragments (i.e., transgenes) in the target genomes can occur (Filipecki and Malepszy, 2006). These position effects can have deleterious consequences on the engineered organisms, but also non-deleterious effects that allow survival of the organisms with no major or apparent phenotypic consequences (Ladics et al., 2015). Nonetheless, in the present study, all the GE crops analyzed were subject to non-directed insertions and we did find significant and unintended phenotypic effects. Currently, GE technology has apparently overcome the problem of position effects through the use of CRISPR/Cas9 (Clustered Regularly Interspaced Short Palindromic Repeats) technologies, which guarantees a more accurate genetic modification through a precise insertion in known locations within the target genomes (Ma et al., 2017). However, the precision of the insertion does not necessarily prevent unintended genetic interactions, such a mutagenesis and pleiotropic effects that could be traced to the phenotypes (Solovieff et al., 2013). The precision of this technology has been recently challenged by research that reveals the presence of high-frequency off-target mutagenesis induced by CRISPR/Cas9 in human and animal cells (Fu et al., 2013; Schaefer et al., 2017).

In maize, phenotypic variation of domGE is reduced and shifted along LD1 compared with domNGE, which is worth noting given the potential implications of introgression (gene flow) with domesticated non-transgenic populations (Quist, 2007) that in turn could decrease the overall variation of domNGE and affect the genetic and cultural repository of diversity that these populations represent. Given the potential of introgression and the risk of affecting the genetic and cultural repositories of biodiversity, the analysis of these unintended effects is extremely valuable for understanding the destiny of hybrids in natural habitats, particularly in the context of environmental biosafety (Arriola and Ellstrand, 1997; Snow et al., 2003).

### Evolutionary Significance of Unintended Phenotypic Changes

In all of the analyzed cases, we can see phenotypic differences among categories (wild, domNGE, and domGE). However, it is worth noting that these differences, although evident through DA and spider charts [**Figure 1**, panels (a) and (b)], are not all statistically significant in the GLM pairwise comparisons between groups [**Figure 1**, (c) panels]. In particular, for sunflower, there is no statistically significant result for the GLM analysis, although differences among populations can be observed in both DA and spider chart analyses. This apparent inconsistency could be rooted in some fundamental reflections about evolutionary processes. For instance, it is known that genetic variation, even if not reflected as statistically significant differences between populations, can be of evolutionary significance (West-Eberhard, 2003). Given that phenotypic variation is directly exposed to natural selection, even small differences that are not statistically significant can have evolutionary consequences for the populations and species. The best example of this is domesticated plants, in which both natural and human selection act on phenotypes, leading to rapid fixation of even rare variants (Zhang et al., 2009; Tang et al., 2010) that statistically could appear as non-significant variation within or among populations. Moreover, this reflection leads to further examination of the finding of these unintended phenotypic effects in the analyzed crops, as it calls attention to the consequences (phenotypic) of genetic introgression events in different economically, culturally, or ecologically relevant crops (domGE→domNGE; domGE→wild). This is particularly important because there is evidence of some of these introgression events [e.g., maize (Quist and Chapela, 2001), rice (Song et al., 2006), and cotton (*Gossypium hirsutum*) (Wegier et al., 2011)]. Although we did not examine consequences of introgressed populations in this study, our results suggest that unintended effects of introgression are possible, and thus need further investigation looking at phenotypic traits that are usually out of scope (such as those associated with fitness), and that can shed more light on the evolution of domesticated (GE and NGE) and wild crop populations.

In addition, the results presented here show how human interventions in plant populations have different consequences in reducing and changing the direction of phenotypic variation to produce food. Historically, these strategies have proven to be effective, but it is worth reflecting on the unintended effects that some interventions can have in these natural resources that might reduce our options to adapt in the future. The reflection on the strategies to follow in this adaptation to future climate change conditions must include a revision of the regulations of crop technologies given the major consequences that this can have in global food security (Smyth and Mchughen, 2008). For example, given the current regulations, it is worth mentioning that the results presented here are in contradiction with the concept of substantial equivalence between NGE and GE crop lines, which argues that given the fact that the lines are isogenic, the resulting lines will only differ in the added trait (Cellini et al., 2004), which can be in fact demonstrated if only the added or target traits are analyzed, but the contrary can happen when looking at non-target traits (Smyth and Mchughen, 2008).

Finally, and in the context of climate change, there is an undeniable urgency to adapt to future uncertain conditions (Wise et al., 2014). However, there is little recognition that some of the current ecosystems (agroecosystems included) may transition to entirely different and unpredictable states, with different goods, services, and natural resources, and that adaptive cycles of decision will be needed in the most ample spectrum of possibilities (Wise et al., 2014). Thus, it is of major importance to preserve options for future decisions, which includes genetic and phenotypic options, in other words biodiversity (Rockstrom et al., 2014).

## Conclusion

The results presented show how human interventions in plant populations have different consequences in reducing and changing the direction of phenotypic variation to produce food. In particular, we found that (1) genetic modification (either by selective breeding or GE) can be traced phenotypically when comparing wild relatives with their domesticated ones (GE and NGE), and (2) the existence and magnitude of the phenotypic differences between domGE and domNGE of the same crop suggest consequences of genetic modification beyond the target trait(s). Further studies documenting phenotypic changes in human modified crops must include as many traits as possible, preferably non-target traits, to design interventions that do not compromise the decision spectrum in the face of trade-offs for adaptation to current versus future conditions.

## Author Contributions

AH-T: designed the research, did the analysis, and wrote the manuscript. AW: designed the research and wrote the manuscript. MB: designed the research and revised previous versions of the manuscript. RL: designed the research. AE: designed the research and wrote the manuscript.

## Conflict of Interest Statement

The authors declare that the research was conducted in the absence of any commercial or financial relationships that could be construed as a potential conflict of interest.

## Footnotes

1 http://www.ddu.unam.mx/index.php/reglamento-general-de-estudios-de-posgrado

## Supplementary Material

DATA SHEET S1 Full dataset of values for all the phenotypic traits included in the meta-analysis profiling rice, canola, maize, sunflower, and pumpkin.
