# Supplementary material for: Domesticated, Genetically Engineered, and Wild Plant Relatives Exhibit Unintended Phenotypic Differences: A Comparative Meta-Analysis Profiling Rice, Canola, Maize, Sunflower, and Pumpkin
Source: Front Plant Sci. 2017 Dec 5;8:2030. doi: 10.3389/fpls.2017.02030 (PMC5723393; doi:10.3389/fpls.2017.02030)

**SUPPLEMENTARY MATERIAL**

**Supplementary table S1**. Coefficients of each trait in the first two Discriminant axes (LD) generated for each crop. The absolute magnitude of the coefficient represents its importance in each Discriminant axis and the sign represents its direction in Discriminant space in Figure 1 (a panels).

| **Crop** | **Traits** | **Coefficients of**  **Discriminants** | |
| --- | --- | --- | --- |
|  |  | LD1 | LD2 |
| **Rice** | Height | 7.04 | 4.38 |
|  | Number of seeds | -3.03 | 4.30 |
|  | Pollen viability | -9.43 | -8.23 |
|  | Days to flowering | 9.31 | 0.909 |
| **Canola** | Number of seeds | 23.7 | -18.6 |
|  | Height | 0.31 | 2.47 |
|  | Days to flowering | 0.62 | 2.46 |
|  | Pollen viability | 2.34 | -1.14 |
| **Sunflower** | Height | -17.2 | -3.97 |
|  | Number of flowers | -1-37 | 2.36 |
|  | Days to flowering | 16.01 | 1.38 |
|  | Number of seeds | -0.78 | 4.44 |
| **Pumpkin** | Number of fruits | 95.9 | -30.5 |
|  | Number of seeds | -63.6 | 56.7 |
|  | Number of flowers | 0.4 | -3.47 |
| **Maize** | Days to flowering | 2.5 | -23.6 |
|  | Height | 8.12 | 15.63 |
|  | Number of seeds | -9.43 | -3.76 |

**Supplementary table S2**. Genetic transformations included in the dataset for each crop.

| **Crop** | **Gene** | **Product** | **Phenotype** |
| --- | --- | --- | --- |
| **Rice** | *Bt* | cry delta-endotoxin | Resistance to lepidopteran insects |
|  | *CpT1* | trypsin inhibitor | Resistance to a wide range of insect pests |
|  | *Bt/CpT1* | cry delta-endotoxin/trypsin inhibitor | Resistance to a wide range of insect pests |
|  | *PPO* | double stranded RNA | Block black spot bruise development |
|  | *Bar* | phosphinothricin N-acetyltransferase (PAT) enzyme | Tolerance to glufosinate herbicide |
|  | *IMI* | modified acetohydroxyacid synthase large subunit (AtAHASL) | Tolerance to imidazolinone herbicide |
|  | *nptII* | neomycin phosphotransferase II enzyme | Metabolism of neomycin and kanamycin antibiotics |
| **Canola** | *Bar* | phosphinothricin N-acetyltransferase (PAT) enzyme | Tolerance to glufosinate herbicide |
|  | *CP4* | 5- enolpyruvulshikimate-3-phosphate synthase (EPSPS) enzyme | Tolerance to glyphosate herbicide |
|  | *Cry1Ac* | cry1Ac delta-endotoxin | Resistance to lepidopteran insects |
|  | *Barnase/bastar* | barnase ribonuclease (RNAse) enzyme/ barnase ribonuclease inhibitor | Male sterility/ restores fertility |
|  | *Bar/nptII* | phosphinothricin N-acetyltransferase (PAT) enzyme / neomycin phosphotransferase II enzyme | Tolerance to glufosinate herbicide / Metabolism of neomycin and kanamycin antibiotics |
|  | *IMI* | modified acetohydroxyacid synthase large subunit (AtAHASL) | Tolerance to imidazolinone herbicide |
| **Sunflower** | *OxOx* | oxalate oxidase | White mold resistance |
|  | *Als* | herbicide tolerant acetolactate synthase (ALS) enzyme | Tolerance to sulfonylurea and imidazolinone herbicides |
|  | *Cry1Ab* | cry1Ab delta-endotoxin | Resistance to lepidopteran insects |
| **Pumpkin** | *ZYMV/WMV* | coat protein of zucchini yellow mosaic potyvirus (ZYMV) / coat protein of watermelon mosaic potyvrus 2 (WMV) | Resistance to ZYMV and WMV |
|  | *CMV/ZYMV/WMV* | coat protein of cucumber mosaic cucumovirus (CMV) / coat protein of zucchini yellow mosaic potyvirus (ZYMV) / coat protein of watermelon mosaic potyvrus 2 (WMV) | Resistance to CMV, ZYMV and WMV |
|  | *ZYMV* | coat protein of zucchini yellow mosaic potyvirus (ZYMV) | Resistance to ZYMV |
| **Maize** | *CP4* | 5- enolpyruvulshikimate-3-phosphate synthase (EPSPS) enzyme | Tolerance to glyphosate herbicide |
|  | *ZMVPP1* | vacuolar H+ pyrophosphatase | Drought tolerance |
|  | *Cry/CP4* | cry delta-endotoxin / 5- enolpyruvulshikimate-3-phosphate synthase (EPSPS) enzyme | Resistance to lepidopteran insects / Tolerance to glyphosate herbicide |
|  | *Cry* | cry delta-endotoxin | Resistance to lepidopteran insects |
|  | *BADH* | betaine aldehide deshidrogenase | Drought tolerance |

**Source:** International Service for the Acquisition of Agri-Biotech Applications (ISAAA) (<http://www.isaaa.org/gmapprovaldatabase/geneslist/default.asp)>.

**Supplementary Figure S1.** Phenotypic differences between wild and domesticated with and without genetic engineering in five crops. Results of the analyses performed without management of outliers. In all panels: a) Discriminant Analysis (DA), b) Spider-Chart of the means of each analyzed trait, and c) results of pairwise comparisons through with Generalized Linear Model (GLM). In all c) “DomPp” = both domesticated populations (GE and NGE). In all panels: “blue” wild relatives, “red” domesticated without genetic engineering and “green” domesticated with genetic engineering. P-value: * < 0.01, ** < 0.001, *** < 0.0001.


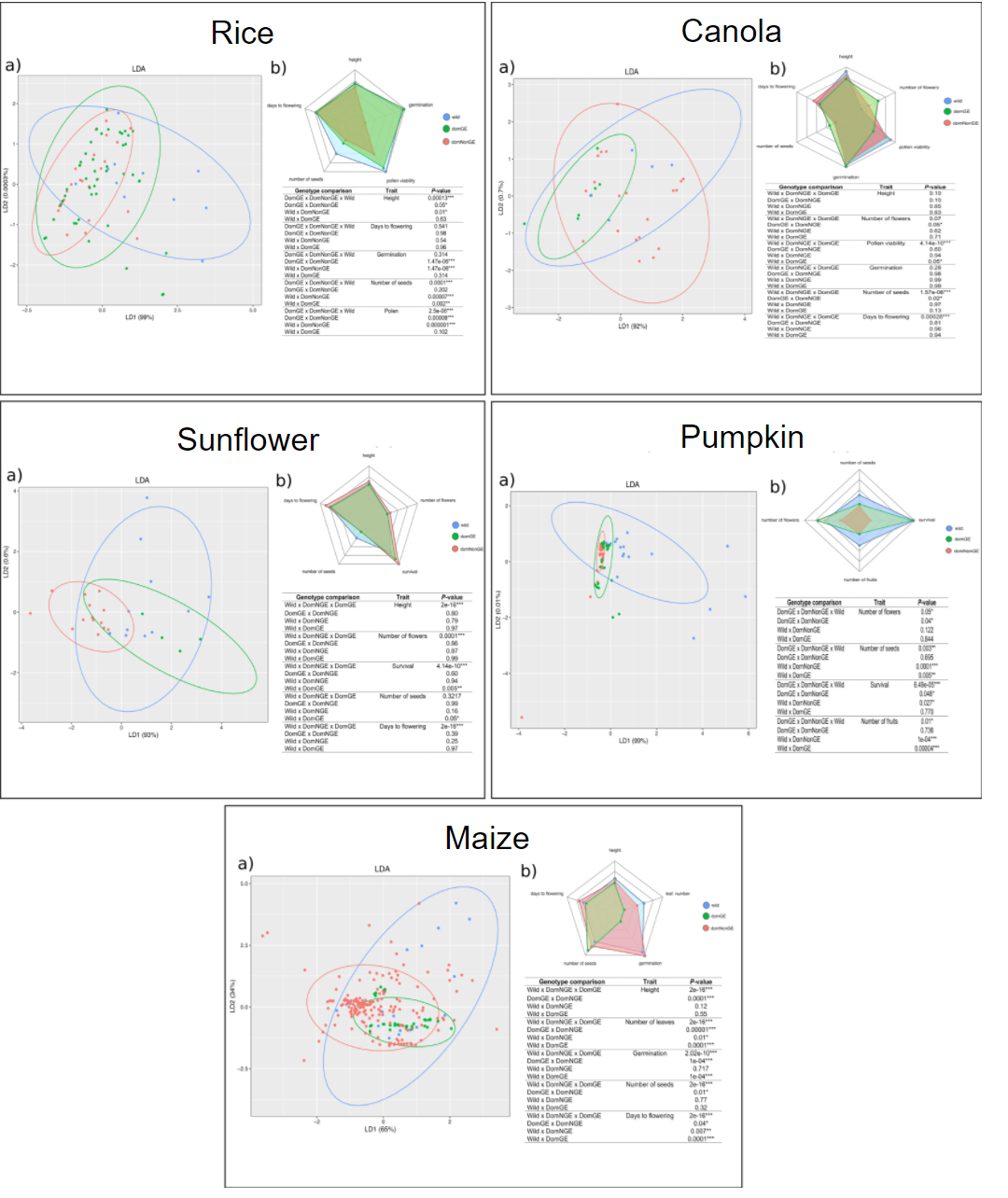

Supplement: Supplementary file 2 [file Table_1.docx]
